# Machine Learning in Intelligent Video and Automated Monitoring

**DOI:** 10.1155/2015/570145

**Published:** 2015-04-09

**Authors:** Yu-Bo Yuan, Gao Yang David, Shan Zhao

**Affiliations:** ^1^Department of Computer Science and Engineering, East China University of Science and Technology, Shanghai 200237, China; ^2^School of Science, Information Technology, and Engineering, Federation University Australia, Mount Helen, VIC 3350, Australia; ^3^Department of Mathematics, University of Alabama, Tuscaloosa, AL 35487-0350, USA

## 1. Introduction

The primary goal of this special issue is to showcase cutting-edge research on tracking and identifying objects, analyzing motion, and extracting interesting frames from analog or digital video streams automatically. At the same time, we particularly focus on the efficiency of video surveillance systems and machine learning methods which can be used to analyze video and control the machine automatically. Our aim is to unify the machine learning techniques as an integral concept and to highlight the trends in advanced video intelligence and automated monitoring.

## 2. Contributions and Results

With the developments of computer science, communication technology, and internet engineering, intelligent video surveillance systems have become more and more important in today's life. They can be seen everywhere. Intelligent video surveillance is digital, network-based video surveillance but is different from the general network video surveillance—it is higher-end video surveillance applications. Intelligent video surveillance system can automatically recognize different objects and find anomalies in the monitor screen. Thus, it potentially provides the fastest and best way to alert and provide useful information, which can help security personnel more effectively deal with the crisis. Moreover, intelligent video surveillance system can maximally reduce false positives and false negative phenomena.

**Figure 1:**
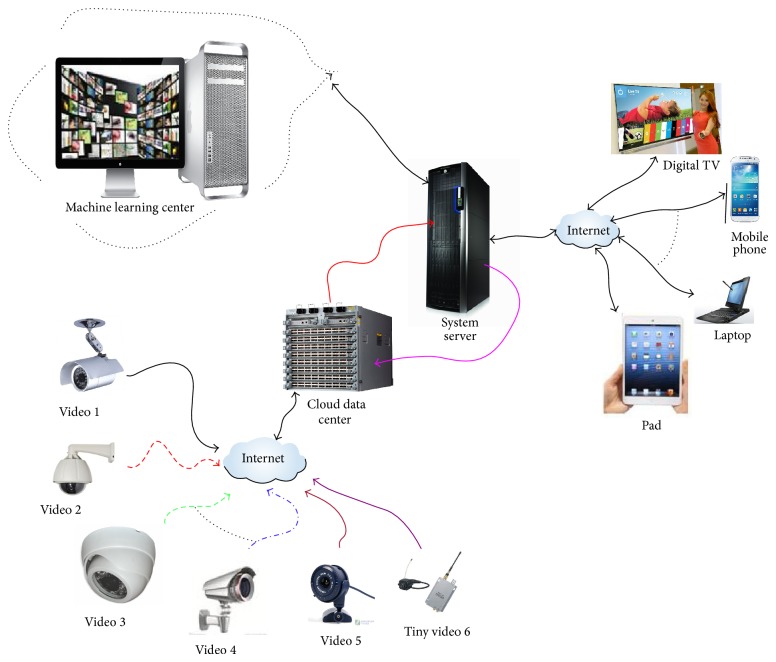
Basic information framework of intelligent video and automated monitoring. More video sources can be collected from video 1 to video 5. In special case, tiny videos are also employed to get video records. The data sets (usually they are big) are submitted to the cloud data center. The services system to handle the videos is the central and important unity. The machine learning system is set up to learn the knowledge or pattern from the special videos according to the users' needs or conditions. In this system, many popular technologies can be employed, such as data mining, manifold learning, kernel learning, image and video processing, and optimization methods and algorithm. In some cases, the machine learning system can transfer the information to users with emails from internet, short messages by mobile communication system, or other dedicated devices (example digital TV sets).

The basic information framework can be found in the illustrated Figure 1.

In this special issue, there were 51 submissions from more than 16 countries including China, the United States, Canada, Germany, France, Australia, Japan, Pakistan, Bangladesh, Korea, Malaysia, South Africa, and Romania. Contributions of the accepted papers are summarized as follows.

Based on the studies on the video data sets, innovative results are reported in some papers. Y. D. Khan et al. proposed a sufficiently accurate method while being computationally inexpensive solution to recognize human actions from videos; H. Fan et al. proposed a novel part-based tracking algorithm using online weighted P-N learning; J. Hariyono et al. presented a good pedestrian detection method from a moving vehicle using optical flows and histogram of oriented gradients (HOG); O. A. Arigbabu et al. presented an effective approach for estimating body related soft biometrics and propose a novel approach based on body measurement and artificial neural network for predicting body weight of subjects and incorporate the existing technique on single view metrology for height estimation in videos with low frame rate; X. Hu et al. proposed a novel local nearest neighbor distance (LNND) descriptor for anomaly detection in crowded scenes; R. Mustafa et al. presented a novel method for detecting nipples from pornographic image contents; J. Zhang et al. set up a new image multilabel annotation method based on double-layer probabilistic latent semantic analysis (PLSA); Z. Wang et al. constructed an accurate pedestrian detection system after combining cascade AdaBoost detector and random vector functional-link net; H. Wang et al. proposed a novel vehicle detection algorithm from 2D deep belief network (2D-DBN) by deep learning framework; J. Li et al. proposed a human action recognition scheme to detect distinct motion patterns and to distinguish the normal status from the abnormal status of epileptic patients after learning video recordings of the movement of the patients with epilepsy; this work is very interesting in the field of health care system of epileptic patients; S. Zhu proposed a new approach to automatically recognize the pain expression from video sequences, which categorize pain as 4 levels: no pain, slight pain, moderate pain, and severe pain.

Some great contributions are devoted to the field of biometrics. Z. Chen et al. presented a novel real-time method for hand gesture recognition using the finger segmentation; D. Li et al. introduced a cost-sensitive learning technology to reweight the probability of test affective utterances in the pitch envelop level and enhanced the robustness in emotion-dependent speaker recognition effectively; H.-M. Zhu and C.-M. Pun proposed an adaptive and robust superpixel based hand gesture tracking system and hand gestures drawn in free air had been recognized from their motion trajectories; Y. Daanial Khan et al. proposed a biometric technique for identification of a person using the iris image.

There are some novel contributions from knowledge management and services selection in the cloud computing. Y. Jiang et al. proposed a tuple molecular structure-based chemical reaction optimization (TMSCRO) method for DAG scheduling on heterogeneous computing systems; Y. Guo et al. proposed a comprehensive causality extraction system (CL-CIS) integrated with the means of category-learning; J. Zhai et al. proposed a novel cost function and improved the discrete group search optimizer (D-GSO) algorithm; H. Zhang et al. proposed a novel web reputation evaluation method quality of service (QoS) information.

